# Altered microRNA expression profiles in the postmortem prefrontal cortex of individuals with alcohol use disorder: a case–control study

**DOI:** 10.1007/s00406-026-02261-7

**Published:** 2026-04-29

**Authors:** Burcu Çaykara Peran, Hani Alsaadoni, Mehmet Zahit Çıracı, Sibel Kuraş, Halime Hanım Pençe, Yalçın Büyük, Bekir Erdoğan, Sadrettin Pençe

**Affiliations:** 1 Department of Medical Biology, Hamidiye Faculty of Medicine, University of Health Sciences, Mekteb-i Tıbbiye-i Sahane (Hamidiye), Kulliyesi Selimiye Mah. Tıbbiye Cad. No:38, 34668 Uskudar, Istanbul Turkey; 2 Department of Medical Biology, International School of Medicine, University of Health Sciences, Istanbul, Turkey; 3 Department of Medical Biochemistry, Hamidiye Faculty of Medicine, University of Health Sciences, Istanbul, Turkey; 4 Department of Forensic Medicine, Faculty of Medicine, Istanbul University-Cerrahpaşa, Istanbul, Turkey; 5 Department of Medical Services and Techniques, Hamidiye Vocational School of Health Services, University of Health Sciences, Istanbul, Turkey; 6 Department of Physiology, Faculty of Medicine, Istanbul Medeniyet University, Istanbul, Turkey

**Keywords:** microRNA, Prefrontal Cortex, Postmortem, Alcohol Use Disorder

## Abstract

**Background:**

MicroRNAs are critical regulators of neuronal plasticity, synaptic signaling, and stress response, and their dysregulation has been implicated in substance use disorders. However, postmortem evidence on their involvement in Alcohol Use Disorder (AUD) remains limited. We aimed to investigate the expression patterns of miR-132, miR-133b, miR-140, miR-181a, miR-190, and miR-212 in the prefrontal cortex of individuals with AUD.

**Methods:**

Prefrontal cortex tissues were obtained postmortem from 30 individuals with documented AUD and 30 age- and sex-matched non-alcohol users. Total RNA was isolated, reverse-transcribed into cDNA, and analyzed by quantitative real-time PCR. Relative expression levels were calculated using the 2^−ΔΔCT^ method, with U6 RNA as endogenous control.

**Results:**

Individuals with AUD exhibited increased expression of miR-133b (fold change: 4.4, *p* = 0.035), and miR-212 (fold change: 1.57, *p* = 0.006), while miR-132 (fold change: 3, *p* < 0.001) and miR-190 (fold change: 2.63, *p* = 0.003) were markedly downregulated compared with controls.

**Conclusions:**

The differential expression of these miRNAs suggests their involvement in neuronal signaling pathways underlying AUD, particularly those linked to synaptic plasticity and stress-related transcriptional regulation.

## Background

Alcohol is one of the earliest discovered psychoactive substances, and Alcohol Use Disorder (AUD) has been documented throughout human history. AUD is characterized by impaired control over alcohol intake and continued consumption despite awareness of harmful consequences, reflecting its chronic relapsing nature and strong neurobiological underpinnings [1]. Globally, alcohol-related disorders are a major public health concern, contributing to approximately 2.5 million deaths annually [2, 3]. Alcohol exerts profound effects on the central nervous system by altering molecular signaling and transcriptional regulation in multiple brain regions. In the context of addiction, dynamic changes in gene expression and regulatory interactions occur across specific neural circuits, particularly those governing reward, stress response, and cognitive control. MicroRNAs (miRNAs), short non-coding RNA molecules that regulate gene expression post-transcriptionally by binding to target messenger RNAs, play a critical role in these processes. By promoting mRNA degradation or inhibiting translation, miRNAs fine-tune neuronal signaling, synaptic plasticity, and neuroadaptive responses to addictive substances [4–6].

Several miRNAs have been implicated in alcohol- and drug-related neuroadaptations, including miR-132, miR-133b, miR-140, miR-181a, miR-190, and miR-212. These molecules are known to influence dendritic spine morphogenesis, synaptic remodeling, and addiction-related pathways in both experimental models and human studies [7–13]. However, postmortem evidence of their expression patterns in the human prefrontal cortex—a region critically involved in decision-making, inhibitory control, and addictive behavior—remains scarce. The prefrontal cortex (PFC) is a core node within addiction-related neurocircuitry, subserving executive functions including decision-making, inhibitory control, and behavioral flexibility. In AUD, structural and functional alterations in prefrontal neuronal circuits are associated with impaired top–down regulation, thereby contributing to compulsive alcohol seeking and vulnerability to relapse [14–16]. Postmortem prefrontal cortex tissue obtained from individuals with AUD enables direct characterization of brain-specific molecular signatures linked to chronic alcohol exposure, minimizing inferential limitations inherent to peripheral biomarker studies.

In this study, we investigated the expression levels of six candidate miRNAs (miR-132, miR-133b, miR-140, miR-181a, miR-190, and miR-212) in postmortem prefrontal cortex tissues of individuals with a history of AUD compared with non-alcohol users. We conducted an exploratory investigation using human postmortem brain tissue to generate evidence relevant to cortical molecular alterations associated with AUD and their potential regulation by selected miRNAs. Based on our findings, we interpret these miRNAs as candidate contributors to AUD-related neuropathological processes rather than validated biomarkers. Their possible utility as biomarkers is therefore considered preliminary and warrants further evaluation in future translational and longitudinal studies.

## Materials and methods

### Case selection and tissue collection

Individuals with AUD were identified through close relative interviews and review of available medical and forensic records. Long-term alcohol consumption was defined as chronic, regular intake consistent with harmful or addictive use for at least 5 years prior to death. Documented clinical diagnoses of AUD or medical records demonstrating alcohol dependence according to DSM-5 criteria were used to support case classification. Control individuals did not have a history of documented AUD or harmful alcohol consumption according to medical records and family reports. Cases with documented neurological or psychiatric disorders, brain-derived diseases, or significant traumatic brain injury were excluded.

Postmortem prefrontal cortex samples were obtained within 24 h after death to minimize RNA degradation. Immediately after dissection, tissues were snap-frozen in liquid nitrogen and subsequently stored at − 80 °C until further processing. A total of 30 individuals with a history of AUD (alcohol group) and 30 individuals without alcohol use (control group) were included.

### RNA isolation and quality control

**Fig. 1:**
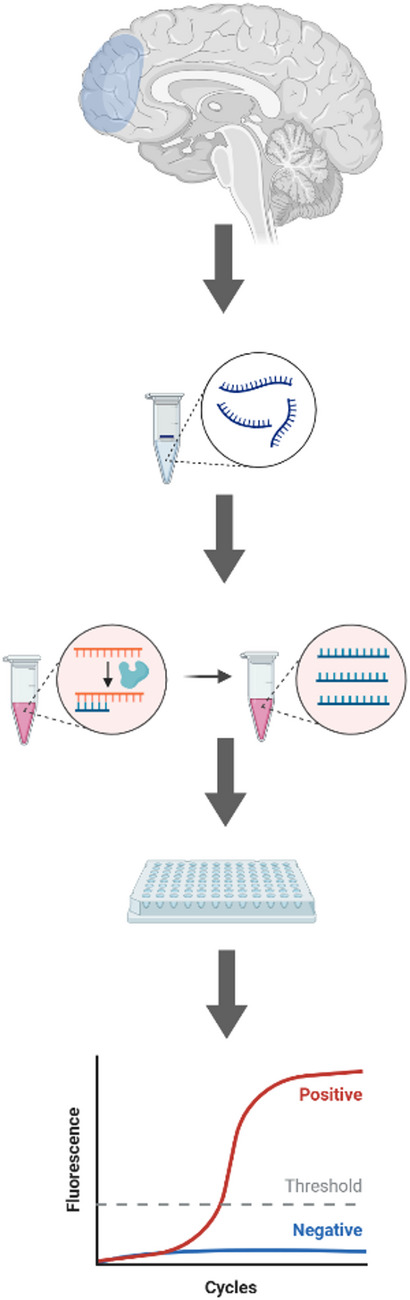
Schematic workflow of candidate microRNAs in the prefrontal cortex

Total RNA was extracted from ~ 30 mg prefrontal cortex tissue sections using QIAzol Lysis Reagent (QIAGEN, Hilden, Germany), following the manufacturer’s instructions. RNA purity and concentration were assessed spectrophotometrically at 260/280 nm using a NanoDrop 2000 instrument (Thermo Fisher Scientific, Wilmington, DE, USA). Samples with A260/A280 ratios between 1.8 and 2.0 were considered acceptable. Figure 1 shows the schematic workflow of the candidate microRNAs investigated in the prefrontal cortex.

Postmortem prefrontal cortex tissue samples were collected, total RNA was extracted, reverse-transcribed into cDNA, and analyzed by quantitative real-time PCR (qRT-PCR). Amplification curves were used to determine relative expression levels. Figure 1 is created in BioRender. Çaykara Peran, B. (2026). https://BioRender.com/izkgcwl.

### Reverse transcription and quantitative real-time polymerase chain reaction (qRT-PCR)

Extracted microRNAs were reverse transcribed into complementary DNA (cDNA) using the miScript II RT cDNA Synthesis Kit (QIAGEN, Hilden, Germany) in a 20 µL reaction volume. Reverse transcription was performed on a thermal cycler under the following conditions: 60 min at 37 °C, followed by enzyme inactivation at 95 °C for 5 min.

Quantitative real-time PCR (qRT-PCR) was carried out using the miScript SYBR Green PCR Kit (QIAGEN, Hilden, Germany) together with specific miScript Primer Assays for each target miRNA (QIAGEN). Amplification reactions were run on an ABI PRISM™ 7000 Sequence Detection System (Applied Biosystems, Foster City, CA, USA). U6 was used as the endogenous control for normalization of miRNA expression levels. The selection of U6 was based on its widespread use in miRNA quantification studies and its reported stability in human brain tissue [17, 18]. Prior to differential expression analysis, the stability of U6 expression was evaluated across all samples and no statistically significant difference found in U6 Ct values (*p* > 0.05). Each sample was analyzed in triplicate to ensure reproducibility. The cycling conditions were as follows: initial activation at 94 °C for 15 min, followed by 40 cycles of denaturation at 94 °C for 15 s, annealing at 55 °C for 30 s, and extension at 72 °C for 30 s. Melting curve analyses were performed to confirm specificity of amplification.

### Statistical analysis

Relative expression levels of target miRNAs were calculated using the 2^−ΔΔCT^ method [19]. Data analysis was performed with SPSS version 22.0 (IBM Corp., Armonk, NY, USA). Normality of data distribution was assessed with the Kolmogorov–Smirnov test. For normally distributed variables, comparisons between groups were performed using Student’s t-test; for non-normally distributed variables, the Mann–Whitney U test was applied. Correlation analyses between miRNA expression and duration of alcohol use were conducted using Spearman’s rho test. A two-tailed p-value < 0.05 was considered statistically significant.

## Results

A total of 60 postmortem prefrontal cortex samples (30 AUD, 30 controls) were analyzed. Demographic characteristics of the study cohort are summarized in Table 1. Two AUD samples were excluded from downstream analyses due to poor amplification quality.

**Table 1:** Demographic characteristics of AUD and control groups

Variable	AUD (*n* = 28*) mean ± SD	Controls (*n* = 30) mean ± SD	*p*-value
Age (years)	51.3 ± 12.7	45.9 ± 15.3	0.202
Age at onset of alcohol use (years)	27.3 ± 7.54	–	–
Duration of alcohol use (years)	24.9 ± 10.75	–	–

*AUD* Alcohol Use Disorder, *SD* standard deviation

**Fig. 2:**
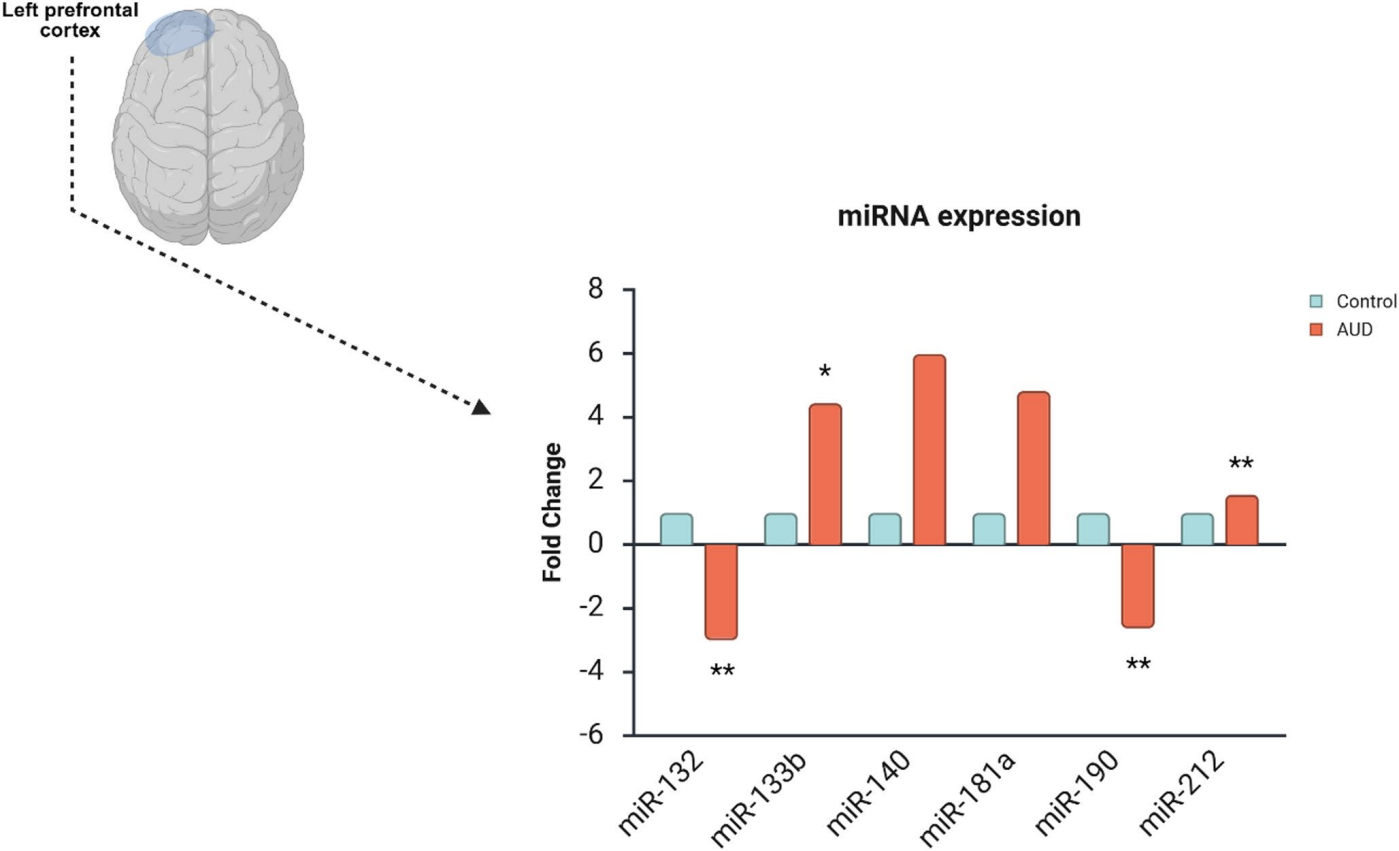
Expression profiles of candidate microRNAs in the prefrontal cortex of individuals with AUD

Expression analysis revealed differential regulation of four candidate miRNAs in AUD compared with controls (Table 2; Fig. 2). Specifically, miR-133b (4.44-fold, *p* = 0.035), miR-181a (4.82-fold, *p* = 0.05), and miR-212 (1.57-fold, *p* = 0.006) were significantly upregulated. miR-140 displayed the largest relative increase (5.98-fold) but did not reach statistical significance (*p* = 0.511). In contrast, miR-132 (0.33-fold, *p* < 0.001) and miR-190 (0.38-fold, *p* = 0.003) were significantly downregulated in the AUD group. Correlation analyses between miRNA expression levels and duration of alcohol use did not yield significant associations (data not shown).

**Table 2:** Fold change and p values of the microRNAs

	miR-132	miR-133b	miR-140	miR-181a	miR-190	miR-212
2^−ΔΔCT^	0.33	4.44	5.98	4.82	0.38	1.57
P value	**< 0.001**	**0.035**	0.511	0.05	**0.003**	**0.006**

p < 0.05

Relative fold-change of four candidate miRNAs (miR-132, miR-133b, miR-140, miR181a, miR-190, and miR-212) in the prefrontal cortex of AUD cases compared to non-alcohol users controls. Data are expressed as fold-change values calculated using the 2^−ΔΔCT^ method. Significant upregulation was observed for miR-133b, and miR-212, whereas miR-132 and miR-190 were downregulated (*p < 0.05; **p < 0.01). No statistically significant differences were observed in miR-140 and miR-181a expressions (*p* > 0.05). Figure 2 is created in BioRender. Çaykara Peran, B. (2026). https://BioRender.com/tnqjml7.

## Discussion

In this study, we observed significant upregulation of miR-133b and miR-212 and downregulation of miR-132 and miR-190 in the prefrontal cortex of individuals with AUD. Among these, miR-133b showed the largest relative increase (4.4-fold), whereas miR-132 displayed the greatest decrease (~ 3-fold) compared with controls (*p* < 0.05). These findings highlight differential regulation of miRNAs involved in neuronal plasticity and addiction-related molecular pathways.

MicroRNAs are short non-coding RNAs of 21–23 nucleotides that regulate post-transcriptional gene expression by promoting mRNA degradation or inhibiting translation, thereby modulating a wide range of cellular and physiological processes [20]. The miR-132/212 cluster is particularly relevant to neuronal morphogenesis and synaptic function. Previous studies have shown that miR-132 is associated with circadian rhythm regulation, while miR-212 has been linked to drug-related motivational processes and protective mechanisms against cocaine addiction [21]. For instance, decreased striatal miR-212 expression has been reported in cocaine-conditioned place preference paradigms in mice [22], and Hollander et al. demonstrated that reduced striatal miR-212 signaling increases vulnerability to cocaine addiction [7].

Human studies also support a neuropsychiatric role of this cluster: postmortem brain samples from individuals with schizophrenia revealed dysregulated miR-132 and miR-212 expression in the dorsolateral prefrontal cortex, with inverse correlations to target genes such as tyrosine hydroxylase and phosphogluconate dehydrogenase [23]. Functional studies suggest that miR-212 modulates learning and memory processes, acting as a negative regulator of recall memory [24], while Hansen et al. showed that the miR-132/212 locus influences overall cognitive capacity [25]. In our study, miR-132 expression was markedly reduced, suggesting its involvement in addiction-related pathways, possibly through impaired synaptic plasticity and circadian regulation. By contrast, miR-212 was moderately increased (1.57-fold), which may reflect compensatory regulation or confounding effects related to other neurobiological variables. The divergent regulation of these two miRNAs within the same cluster underscores the complexity of their roles in alcohol-related neuropathology and warrants further mechanistic investigation.

Previous studies have consistently reported downregulation of miR-133b in response to various addictive substances. Morphine exposure suppressed miR-133b expression in zebrafish embryos and immature rat hippocampal neurons [9]. Similarly, Barreto-Valer demonstrated reduced miR-133b levels in the central nervous system of zebrafish at 24 and 48 h post-fertilization following cocaine exposure [26]. Chen et al. also observed decreased hippocampal miR-133b expression in rats subjected to cocaine-induced conditioned place preference [27]. Beyond substance use, diminished miR-133b expression has been implicated in suicide, suggesting its role in neuropsychiatric vulnerability [28]. In contrast to these findings, our study revealed upregulation of miR-133b in the prefrontal cortex of individuals with AUD compared to controls. To our knowledge, the other report linking alcohol and miR-133b described its downregulation in peripheral blood samples from patients with alcoholic cardiomyopathy [29]. The discrepancy may reflect tissue-specific regulation, as circulating RNA profiles often differ markedly from those observed in brain tissue. Moreover, substance-specific effects (alcohol versus opioids or cocaine) and region-dependent neuroadaptations may contribute to the divergent expression patterns.

The role of miR-140 in neuropsychiatric disorders and addiction is complex and context-dependent. While miR-140-3p has been reported to be downregulated in schizophrenia [21], its expression is increased in response to nicotine exposure [8]. In alcohol-related models, upregulated hsa-miR-140-3p was detected in the medial prefrontal cortex of alcohol-dependent rats [30], consistent with findings from methamphetamine self-administration paradigms, where Du et al. observed elevated miR-140 expression in the prefrontal cortex [31]. Furthermore, miR-140-3p upregulation has been reported in the blood of patients with major depression and bipolar disorder [32, 33]. Although our study showed a 6-fold upregulation of miR-140 in the prefrontal cortex of individuals with AUD, the statistically insignificant distribution of this microRNA suggests that it may not play an active role in the neuroadaptive processes of AUD.

miR-181a has been implicated as a regulator of dopaminergic and glutamatergic signaling. Its expression is induced by dopamine receptor activation and psychostimulant exposure, including cocaine and amphetamines, in experimental models [34]. Zhang et al. demonstrated that miR-181a regulates the glutamate receptor subunit GRIA2, and chronic methamphetamine use downregulates miR-181a in the brain [35]. Reduced miR-181a expression has also been reported in suicide victims [28]. By contrast, upregulation of miR-181a was observed in the nucleus accumbens, prefrontal cortex, and hippocampus following two weeks of cocaine administration [13, 36]. In our study, we observed approximately a fivefold increase in miR-181a expression in the prefrontal cortex of individuals with AUD; however, since the data did not reach statistically significant levels, evidence supporting the idea that it may be effective in AUD-related glutamatergic plasticity cannot be provided.

miR-190 plays a regulatory role in neuronal differentiation through modulation of the transcription factor NeuroD. Chronic opioid exposure consistently reduces miR-190 expression: long-term fentanyl treatment downregulated miR-190 [37], morphine administration decreased hippocampal miR-190 expression in rats [38], and altered expression was observed during cocaine extinction training [27]. Additionally, reduced miR-190 levels have been documented in postmortem brain samples of depressed suicide victims [39]. Consistent with these findings, we observed a 2.63-fold downregulation of miR-190 in the prefrontal cortex of individuals with AUD, suggesting that its suppression may contribute to alcohol-related neuronal dysfunction.

Several limitations of this study should be acknowledged. The sample size was relatively modest and factors such as postmortem interval, RNA integrity, agonal state, and cause of death may have influenced RNA quality and expression profiles. Although we identified differential expression of selected candidate miRNAs, functional consequences and downstream target validation were not examined; therefore, their role in alcohol-related neuroadaptations remain premature. Furthermore, peripheral samples (e.g., blood, cerebrospinal fluid) were not analyzed, which limits the translational potential of our findings for biomarker development. On the other hand, this study provides novel data on miRNA expression profiles in the prefrontal cortex of individuals with AUD. By focusing on biologically informed candidate miRNAs implicated in neuroplasticity and addiction-related pathways, the findings offer clinically relevant insights into alcohol-associated neuropathology. These results provide a basis for future research to further elucidate the molecular background of AUD.

## Conclusion

Our findings demonstrate that miR-132, miR-133b, miR-190, and miR-212 are differentially regulated in the prefrontal cortex of individuals with AUD. In contrast, although miR-140 and miR-181a were analyzed due to their previously reported relevance to addiction-related neurobiological processes, no statistically significant differences in their expression were observed in the present study. These miRNAs are closely linked to synaptic remodeling, neurotransmitter signaling, and neuroplasticity, all of which are central to the pathophysiology of addiction. The observed changes in miR-132, miR-133b, miR-190, and miR-212 suggest candidate molecular mechanisms underlying alcohol-related neuropathology and may represent potential biomarkers or therapeutic targets. Future studies should extend these observations by integrating target gene validation, pathway analysis, and functional assays to clarify the precise molecular circuits through which these miRNAs contribute to AUD.

## Data Availability

The data generated in the present study are not publicly available due (Since the samples were obtained from The Council of Forensic Medicine, the data cannot be made public.) to but may be requested from the corresponding author.
